# The role and mechanisms of polycomb repressive complex 2 on the regulation of osteogenic and neurogenic differentiation of stem cells

**DOI:** 10.1111/cpr.13032

**Published:** 2021-03-23

**Authors:** Yangyang Cao, Le Li, Zhipeng Fan

**Affiliations:** ^1^ Laboratory of Molecular Signaling and Stem Cells Therapy Beijing Key Laboratory of Tooth Regeneration and Function Reconstruction Capital Medical University School of Stomatology Beijing China; ^2^ Tsinghua University Hospital Stomatological Disease Prevention and Control Center Tsinghua University Beijing China; ^3^ Research Unit of Tooth Development and Regeneration Chinese Academy of Medical Sciences Beijing China

**Keywords:** differentiation, EZH2, neurogenic, osteogenic, PRC2 complex, stem cells

## Abstract

The stem cells differentiate into osteoblasts or neurocytes is the key process for treatment of bone‐ or neural tissue‐related diseases which is caused by ageing, fracture, injury, inflammation, etc Polycomb group complexes (PcGs), especially the polycomb repressive complex 2 **(**PRC2), act as pivotal epigenetic regulators by modifying key developmental regulatory genes during stem cells differentiation. In this review, we summarize the core subunits, the variants and the potential functions of PRC2. We also highlight the underlying mechanisms of PRC2 associated with the osteogenic and neurogenic differentiation of stem cells, including its interaction with non‐coding RNAs, histone acetyltransferases, histone demethylase, DNA methyltransferase and polycomb repressive complex 1. This review provided a substantial information of epigenetic regulation mediated by PRC2 which leads to the osteogenic and neurogenic differentiation of stem cells.

## Graphical abstract

As the classical form of PRC2, PRC2‐EZH2 complex plays histone H3K27me3 methyltransferase activity in determining stem cells differentiation, with the interaction of ncRNAs, histone acetyltransferases (HATs), histone demethylase (HDMs), and DNA methyltransferase (DNMs), etc. PRC1‐dependent H2AK119ub1 provides recognition binding sites and promotes further deposition of H3K27me3 in PRC2‐EZH2 loci. With HDMs interaction, H3K4me3 also be modified in the target genes of PRC2‐EZH2. PRC2‐EZH2 regulates the key targets such as DLX5, OSX, RUNX2, and β‐catenin causing the osteogenic differentiation and bone formation, and key targets such as ASCL1a, GFAP, and NGN1 leading to the neurogenic differentiation and functional cells formation.

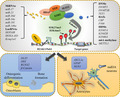

## 1 INTRODUCTION

Adult stem cells are necessary to maintain normal tissue homeostasis in vivo and create a microenvironment (stem cell niche) required for regeneration.^1^, ^2^ There are a lot of bone‐ or neural tissue‐related diseases including osteoporosis, bone defect and malformation, neural tissue injury and defect, neurogenic heterotopic ossification, neurodegenerative diseases with ageing, fracture, injury, inflammation, etc^3^, ^4^, ^5^, ^6^, ^7^, ^8^, ^9^, ^10^ The stem cells could differentiate into osteoblasts or neurocytes to repair the specific tissue and treat these diseases. However, the differentiation process will be affected by different factors, especially the microenvironment. Sometimes stem cells differentiate into the required cell type not very well in microenvironment, which caused undesirable treatment of damaged tissues.^11^ For example, neurogenic heterotopic ossification occurs in 25% cases of spinal cord injury, in which impaired nervous metabolic system causes chaotic bone formation surrounding neural tissue.^12^ This implies that directed differentiation of stem cells requires specific initiation. Therefore, understanding the differentiation regulation of osteogenesis and neurogenesis is crucial for the required tissue repair. The fate determination of stem cells requires a careful balance of genetic and epigenetic programming.^13^ Recent studies have identified that epigenetic regulation determines the stem cell‐specific lineage differentiation.^14^, ^15^, ^16^ Epigenetic mechanisms can maintain the long‐term regulated effects of gene expression in response to environmental stimulation.^16^, ^17^, ^18^, ^19^ Therefore, understanding epigenetic regulation in osteogenic and neurogenic differentiation is of certain guidance for subsequent research and is crucial for promoting the differentiation efficiency and regeneration effect of bone or neural tissue.

Polycomb group complex (PcGs)‐mediated chromatin leading to epigenetic repression is an important regulation mechanism, which caused the dynamic change of gene expression profiles during the differentiation and maturation of stem cells.^20^, ^21^ PcGs‐mediated H3K27me3 is thought to inhibit inappropriate or premature differentiation and may play a key role in determining lineage differentiation of stem cells.^22^, ^23^ Human PcGs mainly consist of two subtypes, the polycomb repressive complex 1 and 2 (PRC1 and PRC2).^22^ PRC1 affects chromatin compactness and can block the transcriptional elongation of RNA polymerase II, thus mediating heterochromatin inhibition.^23^, ^24^ PRC2, through its core component, enhancer of zeste homologue 2 (EZH2), keeps gene silencing by maintaining the silent form of histone H3 Lys27 (H3K27).^25^, ^26^ These two complexes are synergistic, and PRC1 function depends on the PRC2.^27^ And PRC2‐modified H3K27me3 is specifically recognized and bound by factors such as the PRC1 subunit CBX7 that further helps maintaining long‐term gene silencing.^22^ This review mainly focuses on the role and mechanism of PRC2 with EZH2 as the core unit in the osteogenic and neurogenic differentiation regulation of stem cells.

## 2 CORE SUBUNITS OF PRC2

**FIGURE 1:**
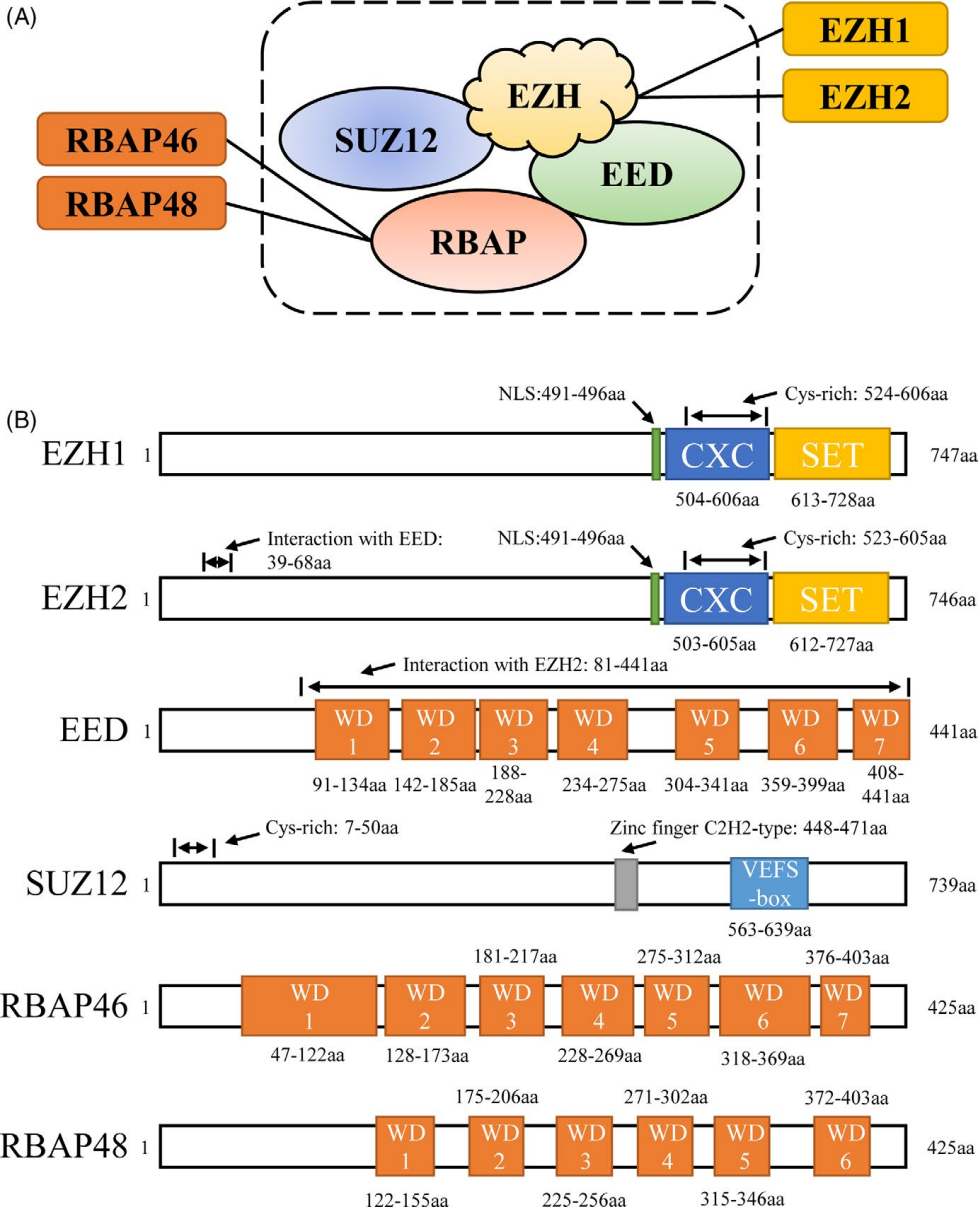
The core subunits of PRC2 in *Homo sapiens*. (A): The core subunits of PRC2. The PRC2 complex mainly contains four core subunits: EZH1/2, SUZ12, EED and RBAP (B): The protein structure of PRC2 core subunits. EZH1/2 is the core subunit of histone H3K27me3 methyltransferase that contains the SET domain and is involved in PRC2 complex formation; SUZ12 mainly maintains the stability of PRC2 complex and assists EZH2 in exerting histone H3K27me3 methyltransferase activity; EED contains the WD repeat function domain and also assists EZH2 in exerting histone H3K27me3 methyltransferase activity; RBAP48 or RBAP46 contain WD repeat function domain involved in the formation, and subsequent histone binding anchoring of PRC2 complex

The core subunits of PRC2 mainly contain four proteins: EZH1 or EZH2, suppressor of zeste 12 (SUZ12), embryo development of the ectoderm protein (EED) and retinoblastoma binding protein (RBAP48/RBBP4 or RBAP46/RBBP7) (Figure 1A).^28^ EZH1/2, involved in PRC2 formation, contains the SET, Homology I/II and CXC domains, which form the catalytic core of histone H3K27me3 methyltransferase. The histone methyltransferase (HMTs) activity of PRC2 mainly depends on the complete SET domain in EZH protein (Figure 1B).^29^ SUZ12 contains the VEFS domain and the C–C–H–H zinc finger structure and mainly maintains PRC2 stability and assists EZH2 in exerting histone H3K27me3 methyltransferase activity (Figure 1B).^30^ EED contains the WD repeat function domain and also assists EZH2 in exerting histone H3K27me3 methyltransferase activity (Figure 1B).^31^ RBAP48 and RBAP46 contain WD repeat function domains and are involved in PRC2 formation and the subsequent binding to histone (Figure 1B).^32^, ^33^

## 3 PRC2 VARIANTS

**FIGURE 2:**
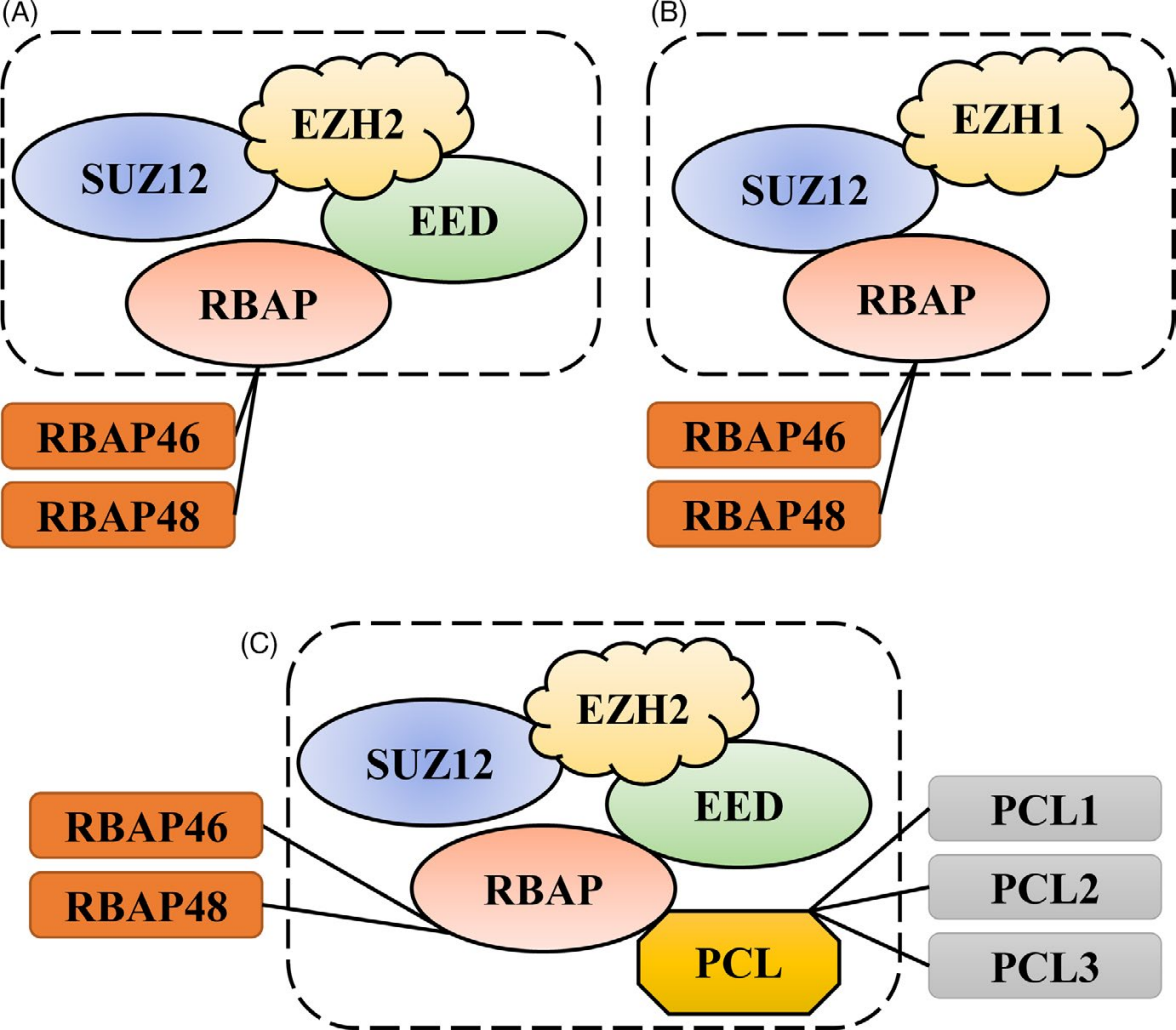
The variants of PRC2 in *Homo sapiens*. (A) PRC2‐EZH2 complex is the classical form of PRC2, this complex mainly contains four core subunits: EZH2, SUZ12, EED, and RBAP and is assembled based on the antedate formation of EZH2‐EED complex. As the classical form of PRC2, PRC2‐EZH2 complex plays the mainly histone H3K27me3 methyltransferase activity. (B) PRC2‐EZH1 complex contain EZH1, SUZ12, and RBAP. Compared with PRC2‐EZH2, its histone H3K27me3 methyltransferase activity is much lower and mainly represses transcription by directly compacting chromatin instead of methyltransferase function. PRC2‐EZH1 seems to switch from a methyltransferase catalytic mechanism to a non‐catalytic mechanism. (C) PRC2‐EZH2‐PCL complex is PcG‐like protein (PCL) binding to PRC2‐EZH2 complex. This complex containing EZH2, SUZ12, EED, RBAP, and PCL. PCL includes PCL1, PCL2, and PCL3, which is not a core subunit and this combination leads complex containing two additional PHD finger structure. PCL is mainly promotes the recruitment and facilitation of classical PRC2‐EZH2 binding to the targeted CpG island and further H3K27me2 to H3K27me3 enrichment

PRC2 plays an important role during epigenetic modification mediated lineage differentiation of stem cells.^13^, ^33^ EZH protein is the core catalytic subunit of PRC2, EZH1 and EZH2 are 65% homologous, and EZH1 is mainly expressed in the adult tissues or the undivided cells, while EZH2 is mainly expressed in the embryogenic or differentiated cells.^28^ Research finds that EZH1 and EZH2 form similar PRC2 complexes but exhibit contrasting repressive roles.^34^ PRC2‐EZH2 complex is the classical form of PRC2, and this complex mainly contains four core subunits: EZH2, SUZ12, EED and RBAP and is assembled based on the antedate formation of EZH2‐EED complex (Figure 2A). As the classical form of PRC2, PRC2‐EZH2 complex plays the mainly histone H3K27me3 methyltransferase activity. PRC2‐EZH2 deletion affects global H3K27me2/3 levels.^34^ Compared with PRC2‐EZH2, PRC2‐EZH1 complex contains EZH1, SUZ12 and RBAP (Figure 2B). Its histone H3K27me3 methyltransferase activity is much lower and mainly represses transcription by directly compacting chromatin instead of methyltransferase function.^34^ PRC2‐EZH1 seems to switch from a methyltransferase catalytic mechanism to a non‐catalytic mechanism.^35^, ^36^ Sometimes EZH1 and EZH2 may have some functional overlap in the PcGs‐dependent H3K27me3 regulation. The study reveals that mouse skin appears pathologic phenotypes only when EZH1 and EZH2 both delete.^21^ And EZH1 colocalizes and preferentially preserves the H3K27me3 on development‐related genes to safeguard embryonic stem cells (ESCs) identity in EZH2^‐/‐^ ESCs. Depletion of EZH1 in EZH2^‐/‐^ ESCs abolishes residual H3K27 methylation and derepresses the target genes.^37^ Study shows that EZH2 deletion results in myelodysplasia, myelodysplastic syndrome (MDS) and myeloproliferative neoplasms (MPNs) development in mice. Only EZH1 deletion does not cause dysplasia in nervous system, which just happens when EZH2 and EZH1 both get deleted. This suggests that EZH1 plays partial compensatory role in hematopoietic system diseases caused by EZH2 deficiency.^38^ PcG‐like protein (PCL) including PCL1, PCL2 and PCL3 can also bind to PRC2 to produce another PRC2 variant and change its nature (Figure 2C).^39^ PCL is not a core subunit and is mainly involved in the recruitment and facilitation of PRC2 binding to the targeted CpG island. This complex contains two additional PHD finger structure and is mainly responsible for PRC2 recruitment and further H3K27me3 enrichment.^28^ In summary, PRC2‐EZH2 complex is the core catalytic complex of H3K27 histone methyltransferase and plays the central role in the epigenetic regulation of PcG complex.

## 4 THE MECHANISMS OF PRC2‐MEDIATED EPIGENETIC MODIFICATIONS

### 4.1 The interaction of non‐coding RNAs and PRC2

The non‐coding RNAs mainly includes microRNAs (miRNAs) and long non‐coding RNAs (lncRNAs). Terminal mesodermal‐specific lineage differentiation of stem cells is reported to be associated with upregulated *miR‐17, miR‐21, miR‐34a and miR‐146a*, along with H3K27me3 repression and reduction in PRC2 subunit SUZ12.^40^ Coinciding with osteogenic markers and the changed gene expression during the osteoblastic induction, several miRNAs such as *let‐7 family, miR‐16, miR‐21, miR‐30 family, miR‐155 and miR‐322* are altered during osteogenic differentiation of mesenchymal stem cells (MSCs) and are designated as OsteomiR. Among these miRNAs, the *miR‐30d* transfectants clarify the context‐dependent targeting of the PRC2 subunit EED.^41^ Another miRNA, miR‐101, is significantly increased during osteogenic differentiation of human bone marrow stromal cells (hBMSCs) and promotes osteogenic differentiation of hBMSCs by targeted EZH2, and EZH2 reverses the osteogenic function of hBMSCs caused by miR‐101.^42^ This suggests that the miRNAs are involved in PRC2‐EZH2‐regulated lineage differentiation of stem cells.^43^

lncRNAs are also regarded as a participant during the lineage commitment and maturation of the stem cell.^44^ Several PcG proteins possess RNA binding activity, which is required for further PcGs binding to DNA. EZH2, the catalytic subunit of PRC2, acts as the key lncRNA nuclear target.^45^ Moreover, at least half of the identified EZH2‐binding lncRNAs are accompanied with a coding gene, either antisense or at the promoter region, suggesting EZH2 involved in local cis‐regulation by lncRNAs.^45^ The lncRNA *CARMEN* has been reported to co‐act with both EZH2 and SUZ12 to regulate the differentiation of cardiac stem cells.^44^ A lncRNA transcribed from the Kcnq1 overlapped transcript 1 (*Kcnq1ot1*) is highly recruited with EZH2 in the mouse brain and enhances neurogenic differentiation of mMSCs.^45^, ^46^ Similarly, systematic analysis reveals the presence of an interaction between *HOTAIR* and PRC2 subunit, *HOTAIR* interacts with PRC2 at HOXD, and knockout of *HOTAIR* reduces H3K27me3 at HOXD site.^47^ It has been shown *HOXA‐AS3* serves as an epigenetic switch of PRC2‐EZH2 histone methylase modification, determining the differentiation of hBMSCs, and is necessary for H3K27me3 modification on the RUNX2 promoter, and knockdown of *HOXA‐AS3* enhances RUNX2 expression and osteogenic ability of hBMSC.^48^ Study found that lncRNA maternally expressed 3 (*MEG3*) could interact with EZH2 and contribute to PRC2‐EZH2 recruitment and promote the transmission and aggregation of PRC2 complex to the specific target genes Glt2, Rian and Mirg and lead to the differentiation of pluripotent stem cells.^49^ It has been shown that the PRC2‐binding lncRNA X inactivation specific transcripts (*XIST*) which containing a 28 bp repetitive element repA, thus providing an example of a specific lncRNA motif that binds to the PcG complex.^50^ Another study has identified a ‘Crab‐claw’ structure which visually has two‐paired 4‐nt loops that act as the molecular basis for lncRNA interaction with EZH2.^51^, ^52^ LncRNAs *Hnf1aos1* and *Gm12840* also contain the same structure that is deemed to accelerate the interaction with EZH2.^45^ In conclusion, the interaction of PRC2‐EZH2 and ncRNAs helps in understanding its epigenetic regulation mechanism, and further studies should investigate whether ncRNAs prevent PRC2‐EZH2 target binding or lead to locus‐specific recruitment (Table 1).

**TABLE 1:** The noncoding RNA interacted with PRC2 during the osteogenic and neurogenic differentiation of stem cells

Noncoding RNAs	Interacted PRC2 Subunits	Stem Cells	Regulated Functions	Ref.
**MiRNAs**				
miR‐17 miR‐21 miR‐34a miR‐146a	SUZ12	Human amniotic fluid isolated stem cells (AF‐MSCs)	Enhances osteogenic differentiation	Zentelytė et al, J cell Biochem. [40]
miR‐30d	EED	Human bone marrow stromal cells (hBMSCs)	Enhances osteoblastic differentiation	Eguchi et al, PLOS ONE. [41]
miR‐101	EZH2	hBMSCs	Promotes osteogenic differentiation	Wang et al, Sci Rep. [42]
**LncRNAs**				
Kcnq1ot1	EZH2	mMSCs	Recruits EZH2 in mouse brain and enhances neurogenic differentiation	Wang et al, Curr Gene Ther. [45]
HOTAIR	EZH2	Human primary stromal cells	Promotes neural crest development	Rinn et al, Cell. [47]
HOXA‐AS3	EZH2	hBMSCs	Represses osteogenic ability	Zhu et al, Oncotarget. [48]

In addition, PRC2 is required to maintain expression of maternal miRNAs and lncRNAs from the *Gtl2‐Rian‐Mirg* locus within the *Dlk1‐Dio3* imprinted gene cluster in mouse ESCs. In the absence of PRC2‐EZH2, the entire *Gtl2‐Rian‐Mirg* locus becomes transcriptionally silent due to gain of de novo DNA methylation at the intergenic differentially methylated regions (IG‐DMRs), a critical cis‐regulatory element that controls expression of maternal *Gtl2‐Rian‐Mirg* locus, and further mechanistic study shows that PRC2 prevents recruitment of Dnmt3 methyltransferases.^49^ These indicate that the action of non‐coding RNAs and PRC2 is reciprocal.

### 4.2 The interaction of DNA, RNA, histone modification genes and PRC2

The interaction of PRC2‐EZH2 subunit with DNA and other histone modifying enzymes has also been reported. PRC2 function and DNA methylation are typically correlated with gene repression. Analysis reveals that PRC2 physically interacts with methyltransferases DNMT3 and reduces recruitment and subsequent DNA methylation at the IG‐DMR, thereby allowing for proper expression of the maternal Gtl2‐Rian‐Mirg locus.^49^ DNA dioxygenases ten‐eleven translocation (TET) is responsible for DNA hydroxymethylation in osteogenic differentiation of hBMSCs. TET1 acts as a repressor of osteogenesis and recruits the co‐repressor proteins EZH2 to osteogenic genes.^53^ The de novo DNA methyltransferase DNMT3B mediates methylation patterns may play an important role in early neurogenesis. Knockdown of DNMT3B shows a loss of H3K27me3 and EZH2 at the promoters of early neural and neural crest specifier genes during the neurogenic differentiation of hESCs.^54^ Moreover, PRC2 function is also correlated with histone modification. Previous study finds that PRC2‐EZH2 increases the H3K27me3 modification level in *WNT1*, *WNT6*, *WNT10a* and *WNT10b* promoters, thus inhibiting the further binding of histone acetyltransferases such as *CBP and p300*. And *CBP‐ and p300*‐modified acetylation and PRC2‐EZH2‐modified H3K27me3 act antagonistically in regulating WNT expression.^55^ The H3K27me3 centred switch between EZH2 and histone lysine demethylation enzyme 6A (KDM6A) determines the hBMSCs differentiation into different lineages. The EZH2‐modified H3K27me3 inhibitory marker is removed by KDM6A during the osteogenic differentiation, which then regulates the conversion of adipogenic to osteogenic differentiation through BMP/TGF and WNT signalling, and the loss of this epigenetic modification leads to development defects of bone tissue.^13^ In addition, SET and MYND domain containing 2 (SMYD2) directly methylates EZH2 at lysine 307 (K307) and enhances EZH2 protein stability by inhibiting its ubiquitination degradation process, which can be relieved by the histone H3K4 demethylase lysine‐specific demethylase 1 (LSD1, also known as KDM1A). SMYD2 and EZH2 collaboratively participate in transcriptional repression of *AXIN2, RASSF1, SIAH1*, etc The dynamic crosstalk between SMYD2 and LSD1‐mediated EZH2 methylation may provide a point for the subsequent involvement of PRC2‐EZH2 in the functional regulation of stem cells.^56^ Researchers observe the interaction between LSD1 and EZH2 proteins in MCF‐7 cells. The interaction between LSD1 and EZH2 stabilizes the binding of LSD1 to *IRF9* promoter, which is a key transcription factor of the interferon pathway.^57^ Then, the balance between H3K4me3 activation and H3K27me3 inhibition may be considered as a rheostat of targeted genes repression.^28^ During human cytomegalovirus (HCMV) infection process, study shows that EZH2 and its regulators Jumonji domain‐containing proteins JARID2 and KDM2B repress *growth factor independence 1 (GFI1)*, *GFI1* acts as a transcriptional repressor of the HCMV immediate‐early promoter (MIEP). EZH2 knockdown delays H3K27me3 in this region which accompanied by the decrease in H3K4me3.^58^ PRC2 functions are also related to RNA modification. Study focuses on METTL3‐mediated m6A modification which plays an important role in regulating neurogenesis and neuronal development through modulating EZH2. MeRIP‐seq analysis reveals that METTL3 mediates N6‐methyladenosine (m6A) levels of EZH2 transcript and METTL3 inhibition downregulates EZH2 and H3K27me3 levels. The defects in neural stem cells (NSCs) proliferation, NSCs neuronal development and the facilitation of METTL3 depletion‐induced NSCs glial lineage differentiation can be rescued by EZH2 overexpression.^59^ In summary, DNA and other histone modifying enzymes play a pivotal role in the regulation of PRC2‐EZH2. Together with different modifying enzymes cross‐talking, the balance between different epigenetic markers modified in the PRC2‐EZH2 targets finally determine the lineage differentiation of stem cells.

### 4.3 The interaction of PRC2‐modified H3K27me3 and PRC1

Chromatin‐modifying activities inherent to PRC1 and PRC2 play an essential role in gene regulation, cellular differentiation and development. In addition to being directly recruited to specific target sites for H3K27me3 modification, PRC2‐EZH2 also functions through other mechanisms. However, no evidence has been found to prove that H3K27 methylation causes gene silencing by changing the interaction between nucleosomes.^28^ The current study notices that H3K27me3 modification in the PRC1 target genes provides recognition of binding sites and promotes the loci inner ring. The cyclization can promote further deposition of H3K27me3 modified PcG loci near, to prevent RNA polymerase activity. Destruction of PRC2‐EZH2 reduces the binding strength of PRC1 to its chromatin target.^28^ Other reports show that PRC1‐dependent H2AK119ub1 leads to prior recruitment of PRC2 and modification of H3K27me3, further leading to the recruitment of PRC1 to target sites. This activity is restricted to variant PRC1 complexes containing KDM2B and is important for the formation of normal polycomb domain in mouse embryonic stem cells and during mouse development.^60^ This suggests a linkage between the PRC subunit proteins and may complete the mechanism of PRC complex‐modified H3K27me3, helping to unravel the PRC2‐EZH2 regulation process, and provides intervention points for more subtle links.

## 5 THE ROLE OF PRC2 IN THE OSTEOGENIC DIFFERENTIATION OF STEM CELLS

It is known that craniofacial skeleton formation is crucially dependent on epigenetic regulation in senior vertebrates.^61^ Previous study shows conditional EZH2 repression causes a strong derepression in *HOX* expression in neural crest cells.^61^ EZH2 deletion in mesoderm and neural crest tissue causes the mortality of embryos, while EZH2 deletion in mesenchyme causes several skeletal abnormalities such as craniosynostosis and bone volume repression.^61^, ^62^ During osteogenic induction in human adipose‐derived stem cells (hADSCs), EZH2 expression is significantly downregulated.^63^ EZH2 inhibition enhances the osteogenic differentiation of hADSCs.^64^ Other studies have reported that conditional knockout of EZH2 inhibits osteogenesis by inducing cell cycle changes in mBMSCs, suggesting that EZH2 plays a bifunctional role during bone formation by suppressing osteogenic lineage commitment, while simultaneously facilitating the proliferative expansion of osteoprogenitor cells.^65^

It has been identified that EZH2‐modified H3K27me3 regulates the lineage specification of stem cells. EZH2 and H3K27me3 are decreased in infected pulp tissue and cells, which were similar to human dental pulp cells (hDPSCs) differentiation.^66^ The expression of EZH2 and H3K27me3 is also decreased during osteogenesis of human dental follicle stem cells (hDFSCs).^67^ EZH2 modifies H3K27me3 of *RUNX2* during osteogenic differentiation of hBMSCs.^48^ Bioinformatic analysis reveals the presence of EZH2 and H3K27me3 in the promoter of osteogenic lineage‐specific transcription factors *FHL1, MX1 and ZBTB16*. EZH2 represses *FHL1, MX1 and ZBTB16* via H3K27me3 regulation, further downregulates *RUNX2* expression and its downstream targets, *osteopontin (OPN)* and *osteocalcin (OCN)* and inhibits the osteogenic differentiation of hBMSCs.^13^ Interestingly, a previous study reports that PRC2‐EZH2‐modified H3K27me3 is significantly increased in mBMSCs of osteoporosis mice model. H3K27me3 inhibitor DZNep effectively reduces PRC2‐EZH2‐modified H3K27me3 levels in the promoters of *WNT1*, *WNT6 and WNT10a*, thereby activating the WNT/β‐catenin signal, and increases bone formation in osteoporosis mice.^68^ Taken together, PRC2‐EZH2 regulates the osteogenic ability of stem cells mainly by modifying H3K27me3 at the promoter of osteogenic lineage‐specific genes or transcription factors. H3K27me3 inhibitors (such as DZNep) or direct deletion of EZH2 (such as genetic modification by virus) can ultimately affect the osteogenic differentiation of stem cells.

Studies have also shown that PRC2‐EZH2 mediates stem cells osteogenesis in vivo. Pre‐treatment with BMP2 and EZH2 inhibitor GSK126 upregulates the osteoblast‐related genes *COL1A1, DLX5, IBSP, OCN, RUNX2 and SP7* expression in hBMSCs on 3D scaffolds in vitro and enhances vascularization but not bone formation in vivo, suggesting that EZH2 might react to multi‐facets of the osteogenesis genes which potentially support the maturation of the osteoblastic lineage.^69^, ^70^ EZH2 and STAT5b‐CA (constitutively active Stat5b) bind to *IRF8* promoter in bone marrow‐derived dendritic cells (hBMDCs) and further repress inflammation and bone loss in prediabetic NOD mice.^71^ But EZH2 inhibition increases bone density in adult mice and alleviates bone loss in ovariectomy osteoporosis mice model.^36^ In addition, GSK126 attenuates bone loss in the ovariectomy osteoporosis mouse model by inhibiting EZH2.^72^ Other study discovers that conditional knockout of EZH2 increases adiposity in bone marrow and causes low trabecular bone phenotype in mice.^65^ EZH2 deficiency potentially activates an adipogenic programme in osteogenic lineage cells. Indeed, fate‐mapping studies have shown that *OSX^Cre+/−^* cells can contain lipid droplets.^65^, ^73^, ^74^ Besides, EZH2 deletion enhances hDPSCs osteogenesis while impairs adipogenesis.^66^ These results hint the role of EZH2 in determining the progression of cell differentiation lineages and ultimately determine the swing between osteogenic and lipogenic differentiation.

For regulation mechanism, researchers discover that EZH2 suppresses the expression of osteogenic genes and ligand‐dependent signalling pathways such as *WNT*, *PTH* and *BMP* to favour the adipogenic differentiation.^63^, ^64^ In other study, EZH2 deletion enhances the expression of *PTH1r and WNT10b* and the osteogenesis in MC3T3 pre‐osteoblasts, and it also increases the BMP‐dependent *Smad1/5* phosphorylation by decreasing H3K27me3 near transcriptional start sites.^36^ WNT signalling core protein, *β‐catenin,* promotes stem cell commitment to osteoblastic lineage and could bind to the EZH2 promoter. *β‐catenin* deletion reduces EZH2 and H3K27me3 levels at the osteogenic loci, while EZH2 inhibition abolishes *β‐catenin*‐promoted osteoblastic commitment.^75^ Other studies show the EZH2 expression and H3K27me3 level decrease during the mineralization of hDPSCs. EZH2 overexpression represses the odontogenic differentiation ability of hDPSCs, while EZH2 mutation in SET methyltransferase domain or knockdown of EZH2 shows that EZH2 loss this repression for hDPSCs differentiation. Mechanically, EZH2 decreases *β‐catenin* by accumulating H3K27me3 on *β‐catenin* promoter and thereby represses the WNT/β‐catenin signalling pathway.^76^ Knockdown of lncRNA MEG3 and EZH2 shows facilitative regulation of hDFSCs osteogenesis by activating the WNT/β‐catenin signalling pathway via epigenetically regulating the H3K27me3 on *WNT1, WNT5A and β‐catenin* promotors.^67^ These results indicate that EZH2 is the key to WNT/β‐catenin effects on stem cells differentiation. Moreover, loss of both EZH1 and EZH2 depresses IGF signalling in chondrocytes and further skeletal growth in mice, suggesting that EZH1 and EZH2 are both required in the PRC2 function.^77^ Recently, a report has shown that cell‐cycle‐dependent protein kinase 1 (CDK1) represses EZH2 expression by inhibiting phosphorylation of EZH2 threonine (Thr) 487 site, which further destroys the combination of SUZ12 and EED; this results in the decrease in PRC2‐EZH2 complex and enhances the osteogenic differentiation of hBMSCs.^78^ Further analysis reports that the expression of EZH2 and SUZ12 and H3K27me3 level were reduced during the osteogenic differentiation of human amniotic fluid‐derived mesenchymal stem cells (hAF‐MSCs).^79^ Here, the core subunits of PRC2 apply interaction during stem cell osteogenesis. The effect of EZH2 can be enriched by interacting with different components of complex.

**FIGURE 3:**
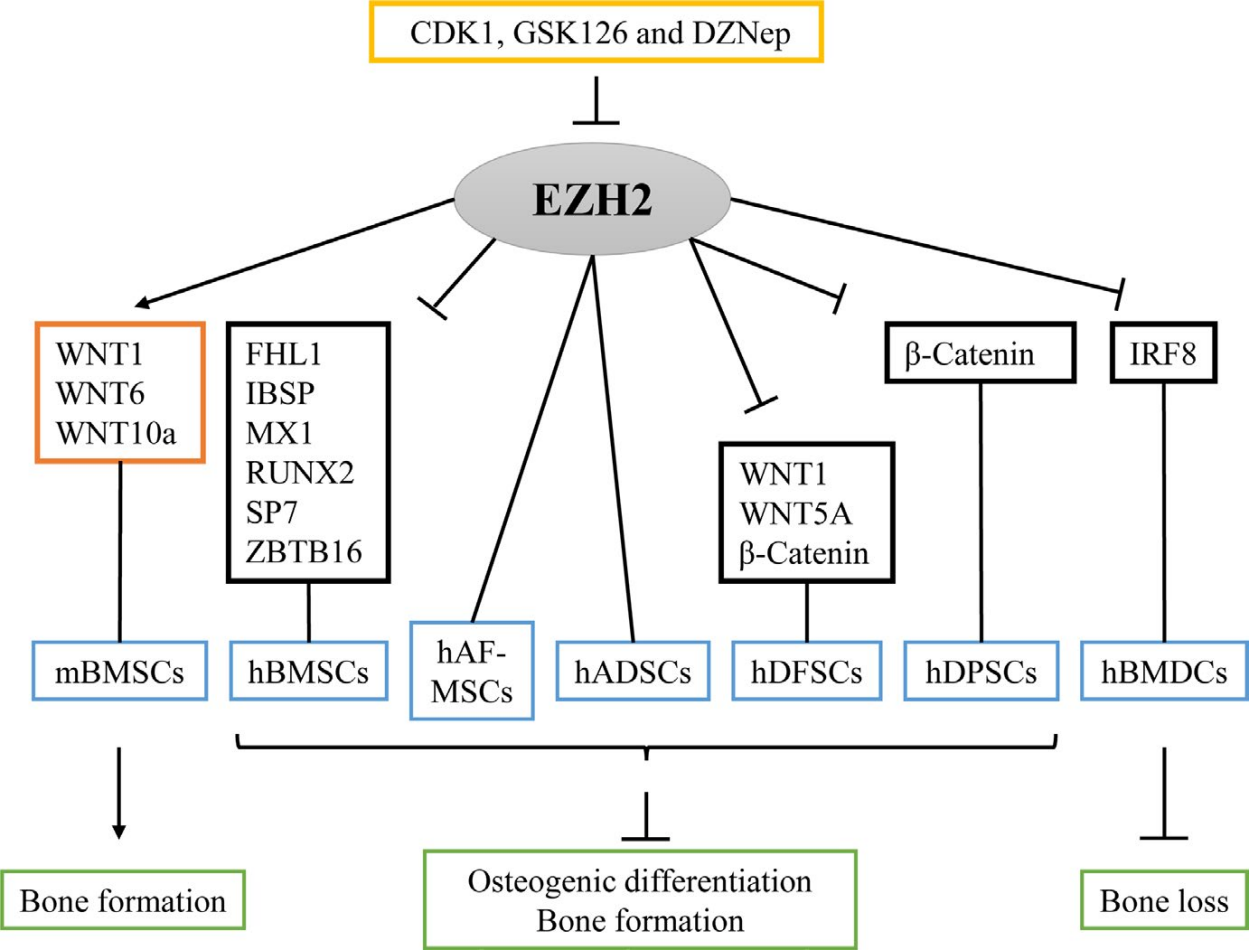
The role of PRC2 core subunits EZH2 in the osteogenic differentiation of stem cell. The core subunit of PRC2, EZH2 activates WNT1, WNT6, and Wnt10a in mBMSCs and increases bone formation of osteoporosis mice. Contrarily, inhibition of EZH2 upregulates the osteoblast‐related genes FHL1, IBSP, MX1, RUNX2, SP7, and ZBTB16 in hBMSCs. With the inhibition of EZH2, the osteogenic differentiation of hADSCs and hAF‐MSCs are enhanced. Knockdown of EZH2 expression shows the decreased H3K27me3 level on the WNT1, WNT5A and β‐catenin promoters, the activation of WNT/β‐catenin signalling pathway and the upregulated osteogenesis of hDFSCs. Similarly, EZH2 deletion also upregulates β‐catenin and enhances osteogenesis of hDPSCs. In pre‐diabetic NOD mice model, EZH2 is found to bind to the IRF8 promoter in hBMDCs and further represses bone loss. And it has been identified that EZH2‐modified H3K27me3 during the lineage specification of stem cells can be effectively reduced by CDK1, GSK126 and DZNep

In conclusion, these discoveries suggest that PRC2‐EZH2 facilitates the early mesenchymal lineage development, but suppresses the late osteogenic lineage differentiation commitment of stem cells to functional osteoblast or osteoprogenitor cells, and serves a bifunctional role during bone formation (Figure 3 and Table 2). The long‐term inhibition of EZH2 does not appear to be beneficial in adolescent or young adult osteoporosis mice model; short‐term or local applications using EZH2 inhibitor in mature or engineered skeletal tissue may accelerate the maturation of differentiated osteoblasts.

**TABLE 2:** The role of PRC2 on the osteogenic differentiation of stem cells

PRC2 subunits	Targeted genes	Stem cells	Regulated function	Ref.
EZH2	FHL1 MX1 ZBTB16	Human bone marrow‐derived mesenchymal stem cells (hBMSCs)	Represses the osteogenic differentiation	Hemming et al, Stem Cells Dev. 2016. [13]
EZH2	RUNX2	hBMSCs and mBMSCs	Represses the osteogenic differentiation	Zhu et al, Oncotarget. 2016. [48]
EZH2	HOX	Neural crest cells	Represses the osteogenic differentiation	Schwarz et al, Development. 2014. [61]
EZH2	‐	Neural crest mesenchyme cells	Inhibits osteogenic and causes skeletal abnormalities	Wyngaarden et al, Development. 2011. [62]
EZH2	‐	Human adipose‐derived stem cells (hADSCs)	Inhibits osteogenic; enhances adipogenic	Dudakovic et al, J Biol Chem. 2015. [64]
EZH2	‐	mBMSCs	Inhibits the osteogenic differentiation; impedes cell cycle progression	Dudakovic et al, J Biol Chem. 2018. [65]
EZH2	‐	Human dental pulp cells (hDPSCs)	Represses the osteogenesis; enhances adipogenesis	Hui et al, J Endod. 2014. [66]
EZH2	WNT1 WNT5A β‐Catenin	Human dental follicle stem cells (hDFSCs)	Represses the osteogenic differentiation	Deng et al, Biochem Biophys Res Commun. 2018. [67]
EZH2	WNT1 WNT6 WNT10a	mBMSCs	Inhibits bone formation and enhances the excessive fat formation	Jing et al, Mol Ther. 2016. [68]
EZH2	IBSP SP7	hBMSCs	Inhibits osteogenic and vascularization	Lui et al, Tissue Eng Part A. 2020. [69]; Dudakovic et al, J Biol Chem. 2020. [70]
EZH2	IRF8	Human bone marrow‐derived dendritic cells (hBMDCs)	Represses bone loss in prediabetic NOD mice	Zerif et al, Int J Mol Sci. 2020. [71]
EZH2	‐	mBMSCs	Causes bone loss in ovariectomy osteoporosis mice	Fang et al, J Immunol. 2016. [72]
EZH2	SP7	Human bone marrow stroma cells	Inhibits osteogenic differentiation; activates adipogenic differentiation	Liu et al, PLOS ONE. 2013. [73]
EZH2	‐	hBMSCs	Inhibits osteogenic differentiation	Sen et al, J Bone Miner Res. 2020. [75]
EZH2	β‐Catenin	hDPSCs	Represses the osteogenic /odontogenic differentiation;	Li et al, J Dent Res. 2018. [76]
EZH2	‐	hBMSCs	inhibits the osteogenic differentiation	Wei et al, Nature Cell Biology. 2010. [78]
EZH2 and SUZ12	‐	Human amniotic fluid‐derived mesenchymal stem cells (AF‐MSCs)	Inhibits the osteogenic differentiation	Glemžaitė et al, Stem Cells Int. 2016. [79]

## 6 THE ROLE OF PRC2 IN THE NEUROGENIC DIFFERENTIATION OF STEM CELLS

One study suggests that EZH2 is responsible for memory, learning, spatial patterning and cognitive functions during hippocampus development in the adult mature brain.^80^ Other study has revealed that PRC2‐EZH2‐modified epigenetic regulation plays a critical role during spinal cord injury and repair.^81^ Recently report shows that PRC2 complex forms decreased during neurogenic differentiation of hESCs.^82^ Neurogenesis begins after the ectodermal cells differentiate into the neuroepithelial cells by neural induction, followed by neurulation.^83^ Neurulation is followed by neural tube formation from neural plates. Then, neural tube is patterned to generate special regions and also gives rise to neural epithelial cells (NECs), mature neurons and glial cells.^84^ After several rounds of proliferation, NECs generate neural progenitor cells (NPCs) or NSCs.^85^ NPCs/NSCs seems to differentiate into neurons firstly and later astrocytes during the neocortical development; this neurogenic‐to‐astrogenic fate switch determines the final repaired neuron generation. Researchers find that EZH2 maintains NPCs self‐renewal and the neurogenic‐to‐gliogenic fate switch of NPCs.^86^ In addition, EZH2 represses the neurogenic and promotes the astrogenic fate transition of NPCs.^87^ Another study has shown that EZH2 expression decreases during the neuronal differentiation and is completely inhibited during the differentiation into astrocytes of mNSCs.^88^ Neuroepithelial cells in ventricular and subventricular zone (VZ and SVZ) produce radial glial cells (RGCs).^87^ The differentiated RGCs produce mature neural cells, while quiescent RGCs form NSCs, which thereafter control SVZ neurogenesis including differentiation into neurons, astrocytes and oligodendrocytes.^89^, ^90^, ^91^ EZH2 is required for NSCs self‐renewal and restricted during the astrogenesis of the cells in the SVZ of adult murine brain.^92^ High level of EZH2 increases oligodendrocyte formation and decreases astrocyte formation from NSCs.^88^ And EZH2 is abundantly expressed in immature neurons, whereas its expression significantly reduced during the maturation of hippocampal neurons.^93^ Moreover, EZH2 regulates the generation of mesodiencephalic dopaminergic (mdDA) neuron and maintains the proper subset identity and the survival of different mdDA neurons during neural development.^94^, ^95^ Knockdown of EZH2 effectively promotes mdDA neuronal differentiation during midbrain development.^96^ Conditional knockout of EZH2 produces ectopic mdDA neurons in the isthmic organizer (IsO) of mice hindbrain.^86^ These suggest that PRC2‐EZH2 participates in the progress of neural differentiation of stem cells and functional neural structure formation by supporting the generation, maturation and maintenance of differential neural lineage.

**FIGURE 4:**
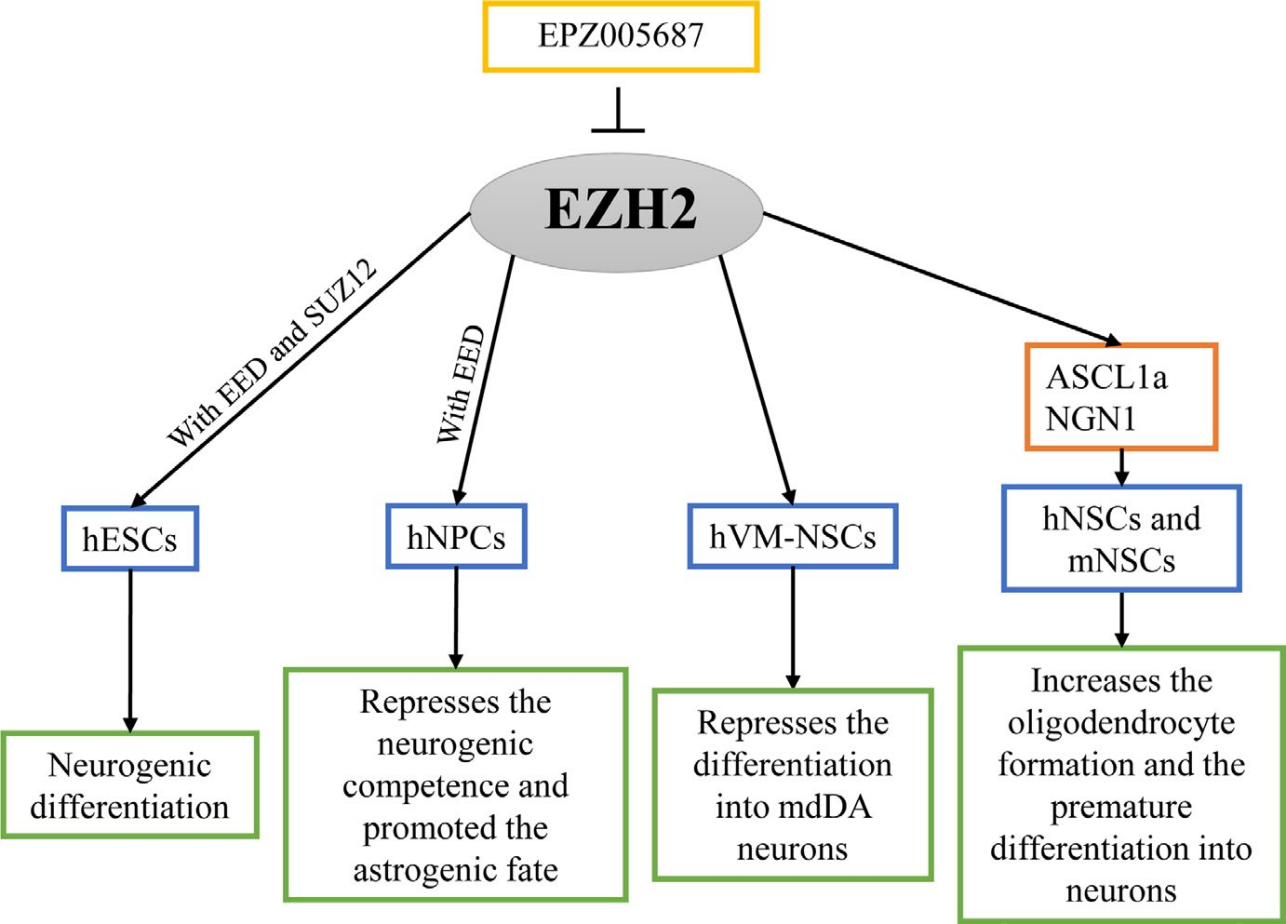
The role of PRC2 core subunits EZH2 in the neurogenic differentiation of stem cells. As the core subunits of PRC2, EZH2 enhances the neurogenic differentiation of hESCs with EED and SUZ12. Respectively, EZH2 represses the neurogenic and promotes the astrogenic fate transition of NPCs with EED. The selective inhibition of EZH2 effectively reduces the H3K27me3 level in hVM‐NSCs and enhances its differentiation into mdDA neurons. Other part, EZH2 increases the oligodendrocyte formation and decreases the premature differentiation into neurons of hNSCs and mNSCs by upregulating ASCL1a and NGN1 expression. And then, PRC2‐EZH2‐modified H3K27me3 can be effectively reduced by EPZ005687

Then, epigenetic mechanism study shows that EZH2 mediates the ubiquitination and proteasomal degradation of aggregated phospho‐serine 129 (*pSer129*) α‐Syn, thus having a neuroprotective anti‐inflammatory potential, oxidative stress reduction and apoptosis prevention.^97^ It is found that EZH2/H3K27me3 levels are elevated in the rat spinal cord after nerve injury.^81^ EZH2 directly represses the cell cycle regulator *p21^WAF1/CIP^* in the chicken spinal cord. EZH2 deletion reduces neural progenitor proliferation and shuffles the neuroepithelium (NE) structure of chick embryo neural tubes, correlating with alteration of the Rho pathway, which is essential for maintaining the neuroepithelial structure.^98^, ^99^ *SOX19b* knockdown decreases the EZH2 upsurge and H3K27me3 at the promoters of *ASCL1a and NGN1*, thereby reducing NSCs proliferation and premature differentiation into neurons.^100^ The selective EZH2 inhibitor EPZ005687 effectively reduces the H3K27me3 level in ventral midbrain‐derived NSCs (VM‐NSCs) and enhances its differentiation into mdDA neurons.^95^ A study has reported a crosstalk between RNA m6A modification and histone modifications. METTL3‐mediated m6A modification plays an important role in regulating neurogenesis and neuronal development through modulating EZH2. MeRIP‐seq analysis reveals that METTL3 mediates m6A levels of EZH2 transcript and METTL3 inhibition downregulates EZH2 and H3K27me3 levels. The defects in NSCs proliferation, NSCs neuronal development and the facilitation of METTL3 depletion‐induced NSCs glial lineage differentiation can be rescued by EZH2 overexpression.^59^ Moreover, the core subunits of PRC2 co‐work during neural differentiation. EED is also essential for spinal cord development and required for neurosphere formation and NSCs proliferation in the SVZ region.^101^, ^102^ EED is highly expressed in the neural tube, and downregulation of EED causes defects in neural tube.^103^ Correspondingly, EED deletion decreases the SUZ12 and H3K27me3 levels in sacral cords of rats with neural tube defects (NTDs).^104^ Inactivation of PRC2‐EZH2 by knockout of EZH2 or EED prolongs neurogenic phase and delays astrogenic phase of NPCs. Further, PRC2‐EZH2 is found to repress the expression of *Neurogenin‐1* in a developmental‐stage‐dependent manner.^105^ The above points indicate that PRC2‐EZH2 acts as the temporal regulator of neurogenic fate in stem cell and inhibits the differentiation of stem cells to functional neurogenic cells, suggesting that it is the key target point for neurogenic regulation (Figure 4 and Table 3).

**TABLE 3:** The role of PRC2 on the neurogenic differentiation of stem cells

PRC2 subunits	Targeted genes	Stem cells	Regulated function	Ref.
EZH2	‐	Human neural stem cells (hNSCs)	Induces neuronal development and glial lineage differentiation	Chen et al, Genomics Proteomics Bioinformatics. 2019. [59]
PRC2 complexes	CDKs	Embryonic stem cells (ESCs)	Enhances the neuronal differentiation	Fukasawa et al, J Biochem. 2015. [82]
EZH2	GFAP	Neuronal progenitor cells (NPCs)	Represses the neurogenic competence and promoted the astrogenic fate	Sparmann et al, EMBO J. 2013. [87]
EZH2	‐	Mouse neural stem cells (mNSCs)	Increases oligodendrocyte formation; decreased astrocyte formation	Sher et al, Stem Cells. 2008. [88]
EZH2	‐	NPCs	Increases mdDA neuron formation during neural development	Wever et al, Front Mol Neurosci. 2018. [95]
EZH2		Ventral midbrain‐derived neural stem cells (VM‐NSCs)	Enhances the differentiation into DA neurons	Hong et al, Stem Cells Dev. 2019. [96]
EZH2	p21^WAF1/CIP^	neural progenitor	Maintains neuroepithelial structure	Akizu et al, Open Biol. 2016. [99]
EZH2	ASCL1a NGN1	NSCs	Promotes neurons differentiation	Li et al, Stem Cell Res Ther. 2019. [100]
EED	‐	NSCs	Promotes neurosphere formation	Sun et al, Cereb Cortex. 2018. [102]
EED	SUZ12	NSCs	Causes neural tube defects in rats	Wang et al, Neurosci Lett. 2010. [104]
EZH2 and EED	‐	NPCs	Prolongs the neurogenic phase; delays the astrogenic phase	Hirabayashi et al, Neuron. 2009. [105]

## 7 CONCLUSIONS

In summary, as the core catalytic component of H3K27 histone methyltransferase, the PRC2 has robust regulatory effects on osteogenic and neurogenic differentiation of stem cells via epigenetic mechanisms, and PRC2‐EZH2 acts as the typical variant form of PRC2 complex. Its regulation function is usually associated with the interaction of ncRNAs, histone acetyltransferases (HATs), histone demethylase (HDMs), DNA methyltransferase (DNMs) and the cross‐talking between PRC2 and PRC1. The information in this review may help to further clarify PRC2‐EZH2 role and the deep modification rules in osteogenic/neurogenic differentiation of stem cells and provides potential targets to regulate the associated function of PRC2‐EZH2. Since the known regulated target of PRC2‐EZH2 and H3K27me3 mainly relies on epigenetic inhibition or direct deletion of EZH2, further study may focus on the core functional fragments and bio‐active modulators of PcGs protein, which may improve the fine regulation of PRC2‐EZH2, control the specific differentiation initiation and transformation of stem cells and enhance the regeneration of bone or neural tissues and the therapy of diseases involving bone or neural tissues.

## CONFLICT OF INTEREST

The authors declare no potential conflicts of interest.

## Data Availability

All data used to support the findings of this study are included within the article.
